# Supplementary material for: A Novel Ginsenoside-Transforming α-L-Rhamnosidase from Bifidobacterium: Screening, Characterization and Application
Source: Biomolecules. 2024 Dec 16;14(12):1611. doi: 10.3390/biom14121611 (PMC11673932; doi:10.3390/biom14121611)
Supplement: Supplementary file 1 [file biomolecules-14-01611-s001.zip › biomolecules-3321145-supplementary.pdf]

Supplementary Materials

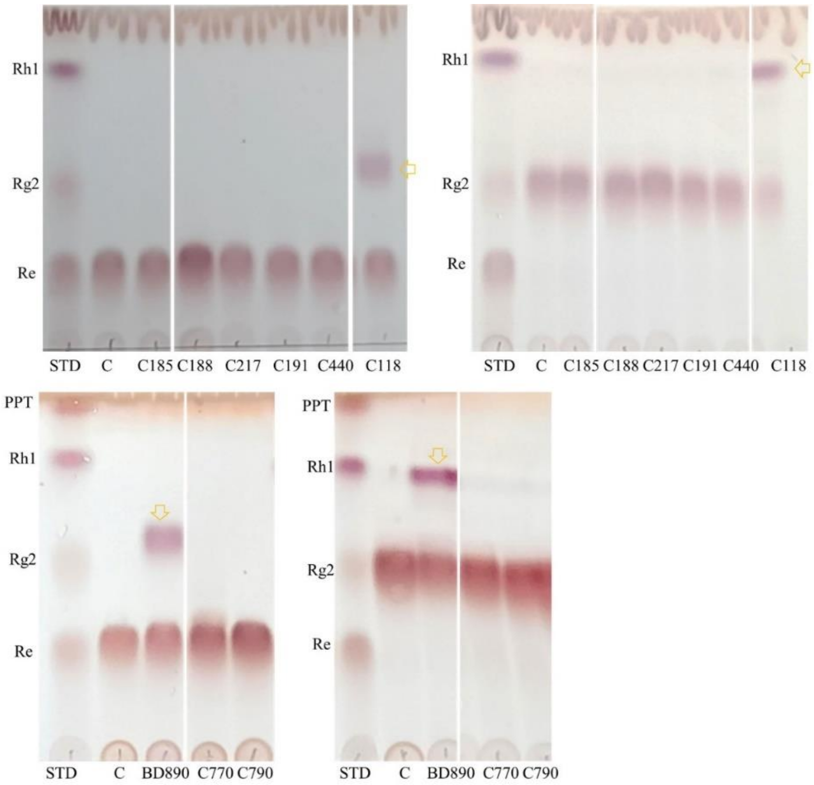

Figure S1. Analysis of the biotransformation of Re and Rg1 by rhamnosidases using TLC. Lanes: STD, ginsenoside standard; C, substrate.

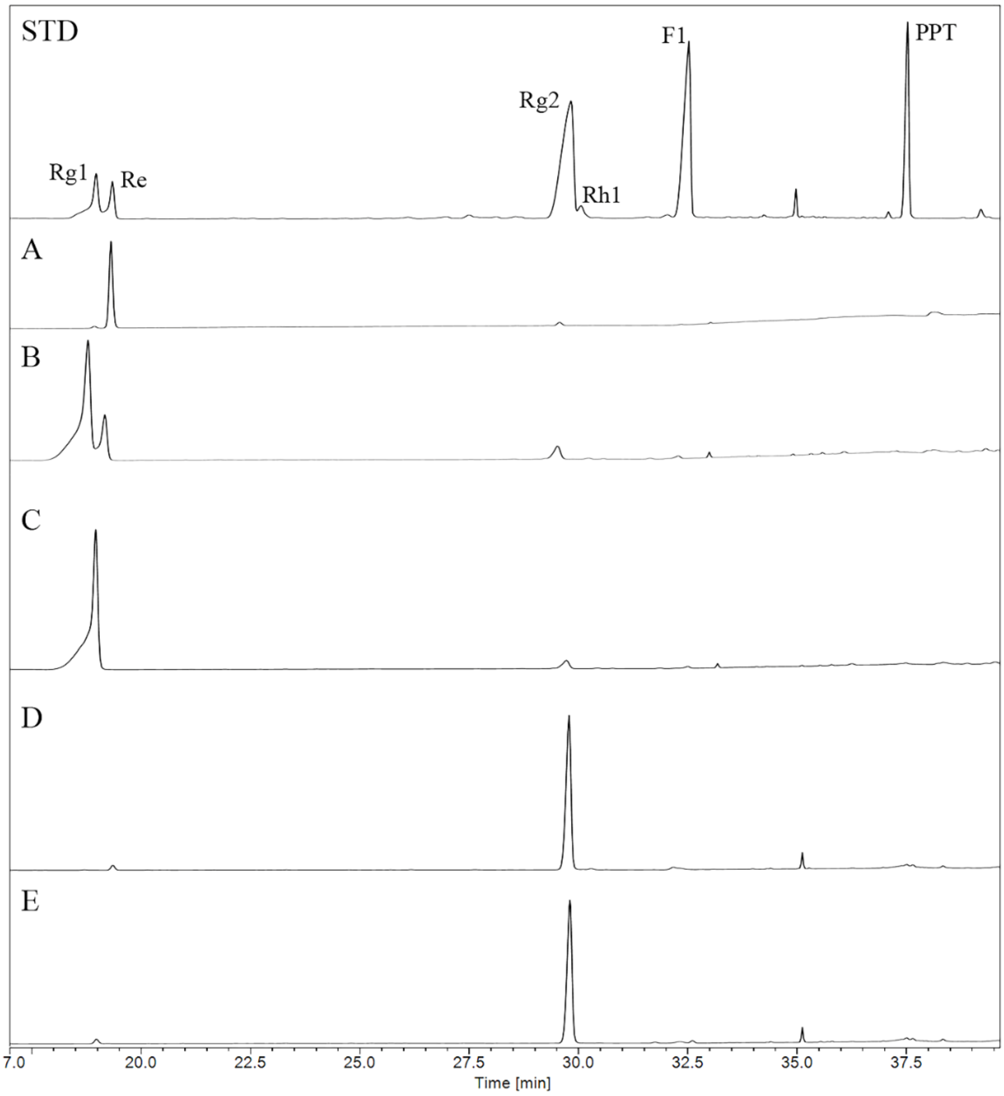

Figure S2. Assessment of ginsenoside Re and Rg2 transformation by recombinant C118 using HPLC. (STD) Ginsenoside standards; (A) Control sample of ginsenoside Re without C118; (B) Reaction mixture after 5 hours of C118 treatment; (C) Reaction mixture after 48 hours of C118 treatment; (D) Control sample of ginsenoside Rg2 without C118; (E) Reaction mixture after 48 hours of C118 treatment.

Table S1. Effects of metal ions and chemical agents on the activity of recombinant C118.

Ions	Relative activity %
β-mercaptoethanol	100.3 ± 2.3
CaCl2	121.4 ± 7.3
CoCl2	99.7 ± 5.2
CuSO4	22.2 ± 4.4
NaCl	99.7 ± 2.5
MgCl2	92.1 ± 1.6
MnCl2	110.2 ± 6.3
EDTA	104.6 ± 3.4
SDS	91.8 ± 5.4
KCl	96.7 ± 4.5
Control	100.0 ± 3.7

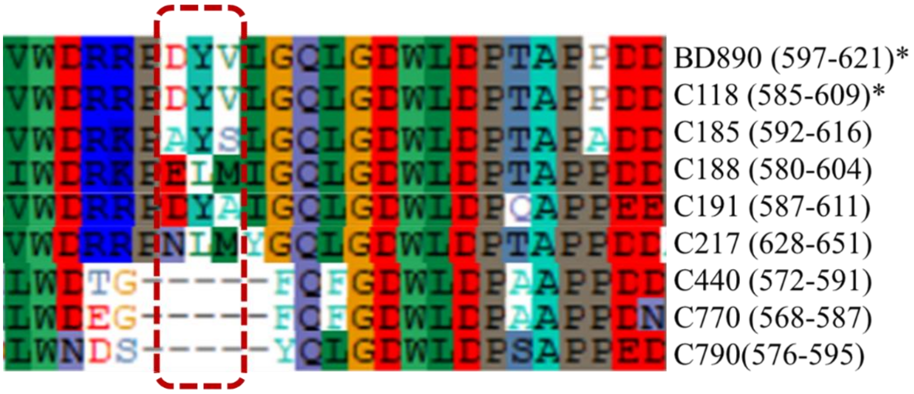

Figure S3. Comparison of conserved sequence motifs in 9 rhamnosidases studied. An asterisk (*) indicates the ginsenoside-transforming rhamnosidases. Key amino acid differences are highlighted with red squares. Multiple sequence alignments of rhamnosidases were conducted using Clustal Omega (1.1.0).

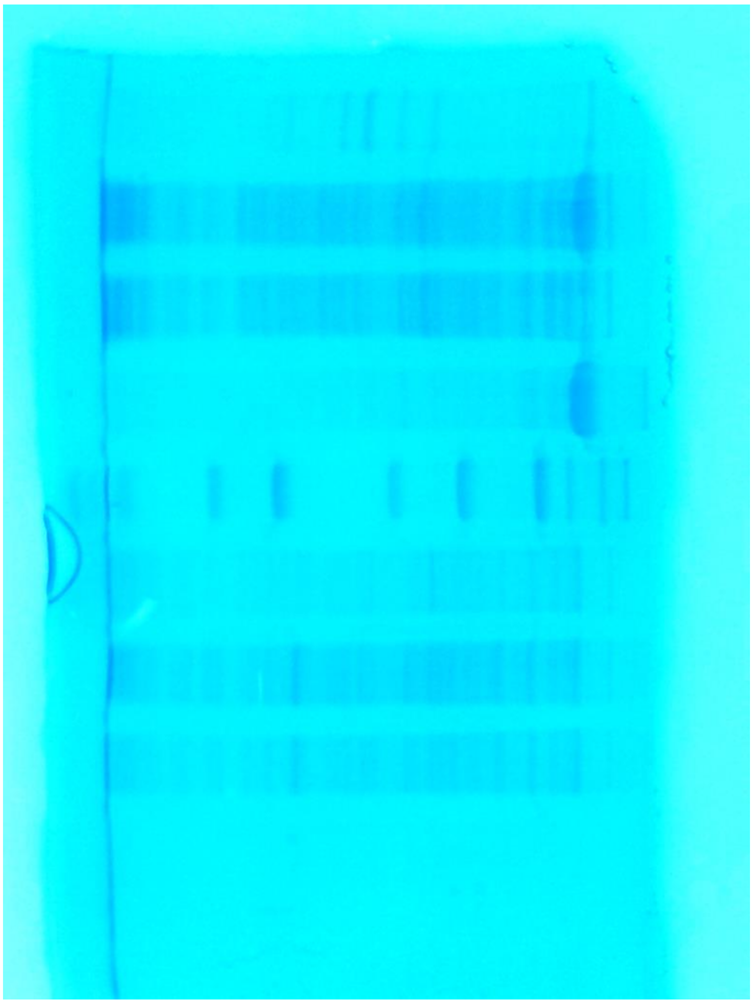

Figure S4. Original image of Figure 3.
